# Nonclinical Sexual Health Support for HIV, Viral Hepatitis, and Other Sexually Transmitted Infections in Gay, Bisexual, and Other Men Who Have Sex With Men: Protocol for a European Community Health Worker Online Survey (ECHOES)

**DOI:** 10.2196/15012

**Published:** 2020-02-18

**Authors:** Nigel Sherriff, Jorg Huber, Nick McGlynn, Carrie Llewellyn, Alex Pollard, Nicolas Lorente, Cinta Folch, Caoimhe Cawley, Oksana Panochenko, Michael Krone, Maria Dutarte, Jordi Casabona

**Affiliations:** 1 School of Health Sciences University of Brighton Brighton United Kingdom; 2 Centre for Transforming Sexuality & Gender University of Brighton Brighton United Kingdom; 3 School of Environment & Technology University of Brighton Brighton United Kingdom; 4 Brighton and Sussex Medical School University of Sussex Brighton United Kingdom; 5 Centre d’Estudis Epidemiològics sobre les Infeccions de Transmissió Sexual i Sida de Catalunya (CEEISCAT) Agència de Salut Pública de Catalunya Badalona Spain; 6 Institut Investigació Germans Trias i Pujol (IGTP) Badalona Spain; 7 CIBER Epidemiología y Salud Pública (CIBERESP) Madrid Spain; 8 Robert Koch Institute Berlin Germany; 9 AIDS Action Europe Berlin Germany; 10 European AIDS Treatment Group Brussels Belgium

**Keywords:** community health worker, ECHOES, Europe, MSM, gay men, HIV, hepatitis, sexual health, sexually transmitted infections, peer support

## Abstract

**Background:**

The term “community health worker” (CHW) can apply to a wide range of individuals providing health services and support for diverse populations. Very little is known about the role of CHWs in Europe working in nonclinical settings who promote sexual health and prevent HIV and other sexually transmitted infections (STIs) among gay, bisexual, and other men who have sex with men (MSM).

**Objective:**

This paper describes the development and piloting of the first European Community Health Worker Online Survey (ECHOES) as part of the broader European Union-funded ESTICOM (European Surveys and Trainings to Improve MSM Community Health) project. The questionnaire aimed to assess the knowledge, attitudes, and practices of CHWs providing sexual health services to gay, bisexual, and other MSM in European settings.

**Methods:**

ECHOES comprises three superordinate domains divided into 10 subsections with 175 items (routed) based on a scoping exercise and literature review, online prepiloting, and Europe-wide consultation. Additional piloting and cognitive debriefing interviews with stakeholders were conducted to identify comprehension issues and improve the clarity, intelligibility, accessibility, and acceptability of the survey. Psychometric properties, including internal consistency of the standardized scales used as part of the survey were examined. The final survey was available to 33 countries in 16 languages.

**Results:**

Recruitment closed on January 31, 2018. Data from 1035 CHWs were available for analysis after application of the exclusion criteria. The findings of the ECHOES survey and the wider ESTICOM project, are now available from the ESTICOM website and/or by contacting the first author.

**Conclusions:**

The findings of this survey will help characterize, for the first time, the diverse role of CHWs who provide sexual health services to gay, bisexual, and other MSM in Europe. Importantly, the data will be used to inform the content and design of a dedicated training program for CHWs as part of the larger ESTICOM project and provide recommendations for MSM-specific strategies to improve sexual health in general and to reduce the incidence and prevalence of HIV, viral hepatitis, and other STIs in particular.

**International Registered Report Identifier (IRRID):**

RR1-10.2196/15012

## Introduction

Individuals who work in community-based settings have an important role to play in sexual health promotion and prevention of HIV and sexually transmitted infections (STIs) among gay, bisexual, and other men who have sex with men (MSM) [1-4]. In the United States [5-7] and elsewhere [8-10], such workers and volunteers are often characterized as community health workers (CHWs) - a workforce that has gained increased recognition, visibility, and legitimacy, and, in the United States at least, is now seen as an essential part of the public health system [11].

In a more global context, CHWs can be an important complement to underresourced health workforces, and thus can potentially be important to increase the availability of and access to health services [12,13]. Indeed, the evidence base regarding the positive contribution CHWs can make in the delivery of population-based health interventions is growing, particularly for child and maternal health, noncommunicable diseases, and infectious diseases [14].

In the countries of the European Union (EU) and European Economic Area (EEA; which includes EU countries as well as Iceland, Liechtenstein, and Norway), it is within the sphere of infectious diseases, specifically HIV and other STIs, that the concept and role of CHWs has recently come to the fore. MSM continue to represent the predominant mode of HIV transmission in the EU and EEA, accounting for 38% of all new HIV diagnoses in 2017 [15]. Although some countries have started to note a decline in HIV incidence among MSM (namely, Belgium, Greece, the Netherlands, Spain, and the United Kingdom), overall rates of HIV diagnoses among MSM continue to increase.

The reasons for MSM being disproportionately affected by HIV and other STIs, including viral hepatitis, are complex and vary along geographical and historical differences of the EU and EEA. Factors include (but are not limited to) the complex interactions between sexual behaviors; STIs; an increased biological vulnerability for HIV infections; social stigma associated with homosexuality; syndemics of mental health issues and substance use and misuse among MSM; structural, psychological, and provider-associated barriers experienced by MSM when accessing sexual health services; a lack of data and research on MSM in many countries; a lack of funding for MSM-targeted HIV and STI prevention and community-based HIV testing; advances in communication technologies and their impact on partner seeking and sexual behavior; and high internal and cross-border mobility (eg, [16]).

Historically, and in addition to the previous list, the public health sector in many European countries was slow in responding to the HIV epidemic (for example, due to conservative legislation around same-sex relationships and cultural and socioeconomic barriers fuelling stigma), leaving a void among other things in prevention and advocacy activities and service development [16]. This void was filled out of necessity by gay communities and nongovernmental organizations (NGOs), particularly in Western Europe, which proactively and progressively developed HIV prevention initiatives, programs, and services targeted to the most affected key populations, including MSM.

Unfortunately, such programs and services have been burdened over the years by insecure funding streams (eg, donations), poor linkage with formal health systems, lack of training and support for workers, fragmentation of purpose and roles, and competition for scarce resources with other actors and/or organizations. Together with a mixed and diverse nomenclature to characterize workers or volunteers (eg, HIV prevention worker, outreach worker, sexual health worker, health promoter, peer counselor, volunteer, health educator), this has led to a somewhat fractured and unstable workforce. For instance, in Europe the term “community health worker” (or “CHW”) is rarely used; instead, a plethora of disparate terms take its place (eg, [17-19]), which vary across organizations and countries. These definitional uncertainties result in a poor understanding of the precise nature of CHW work, practices, roles, knowledge, skills, and needs [3,20].

In 2015, as part of the European Commission’s Health Program 2014-2020, the Consumers, Health, Agriculture and Food Executive Agency (Chafea) issued a tender specification providing an important opportunity to not only strengthen the community response to tackling HIV and other STIs among MSM, but to also raise awareness regarding the persisting legal, structural, political, and social barriers hindering a more effective response to the syndemics of HIV, viral hepatitis B and C, and other STIs among MSM. The tender requested the development of a “behavioural survey for HIV/AIDS and associated infections, and a survey and tailored training [programme] for community-based health workers (CHWs) to facilitate access and improve the quality of prevention, diagnosis of HIV/AIDS, STI and viral hepatitis, and health care services for MSM.” In this tender, the term “CHW” was introduced for the first time, as far as we are aware, to refer to the workforce in Europe that supports the sexual health needs of MSM around HIV, viral hepatitis, and other STIs.

This paper is based on the pan-European 3-year project entitled ESTICOM (European Surveys and Training to Improve MSM Community Health) that was funded via this Chafea tender (no. Chafea/2015/Health/38). ESTICOM (2016-2019) aims to develop (1) a European online survey among MSM (European MSM Internet Survey—EMIS 2017); (2) a European online survey regarding the knowledge, attitudes, practices, and training needs of CHW who support MSM (ECHOES—the European Community Health Workers Online Survey); and (3) a training program for MSM-focused CHWs adaptable for all EU countries.

In this paper, we present the protocol for the ECHOES survey as a core part of the larger ESTICOM project, which is an extensive questionnaire that grappled with definitional complexities of CHWs who support MSM. The overarching aim of the ECHOES survey was to gather data from CHWs to help understand their role, including their knowledge, attitudes, and practices. Ultimately, the information should aid the potential development of the workforce through training, support, and policy development [21].

## Methods

### Design

A quantitative self-report questionnaire (ECHOES) was designed within the European Commission’s funded ESTICOM project. The questionnaire was administered online using the survey tool provided by the Demographix platform.

### Aim and Objectives

The overarching aim of the ECHOES study was to develop a multilingual, Europe-wide online questionnaire capable of assessing the knowledge, attitudes, and practices of CHWs providing sexual health services to gay, bisexual, and other MSM. Specifically, the research objectives were to (1) generate insight about who CHWs in Europe are, what they do, where they do it, and how and why they do it; (2) identify barriers and challenges to CHW activities; (3) identify skill and knowledge gaps and training needs; and (4) generate insights for the development of a dedicated training program for CHWs.

### Study Population

ECHOES is the first survey of its kind in Europe that addresses CHWs who provide sexual health support to gay, bisexual, and other MSM. Therefore, the CHW study population is mostly unknown to researchers, an issue that the ECHOES survey was designed to address. Thus, given the term “CHW” is not well-known or used in Europe, a deliberately broad working definition was developed by the ECHOES development team to define the study population. Following an informal review of the relevant literature, this working definition was achieved through a consensus-based process with consortium partners using elements of the nominal group technique. The nominal group technique is essentially a group process involving problem identification, solution generation, and decision making. It can be particularly useful to ensure all parties are able to contribute and for use when the issue under question is controversial and/or the primary purpose is to come to clarification (rather than resolve differences of opinion). Thus, for ECHOES, a CHW was defined as “Someone who provides sexual health support around HIV/AIDS, viral hepatitis, and other sexually transmitted infections (STIs), to gay, bisexual, and other MSM. A CHW delivers health promotion or public health activities in community settings (not in a hospital or clinic).” [19] In other words, according to our definition, a CHW can be any person working with MSM around sexual health support (paid or unpaid) with or without a medical or health background as long as their work is conducted in community or nonclinical settings. Such a definition was intended to capture not only those more traditionally associated with supporting MSM, such as HIV outreach workers working in gay venues on behalf of NGOs, but also those providing sexual health support in a variety of different sectors (eg, educational, social care, housing, private sector) and in diverse ways.

Detailed plans to engage with the target population and recruit them to the survey were developed by the consortium partner AIDS Action Europe in collaboration with study partners. AIDS Action Europe is a network of national networks, AIDS service organizations, and community-based groups representing 415 NGOs in 47 countries in the World Health Organization (WHO) European Region. Briefly, activities included an initial Europe-wide consultation exercise to generate insight into the most useful communication channels to reach CHWs with 44 responses received from 32 countries (29 from countries eligible to be surveyed). Other strategies to recruit participants included direct mailing and emailing (eg, using translated email templates), website news items shared with pan-European HIV/AIDS organizations, paid social media promotion (Facebook), personal and professional contacts (eg, via events such as the HIV/AIDS, TB and Hepatitis Civil Society Forum), interviews and case studies published online “showcasing” the survey in specific countries, as well as a European webinar and marketing activities at relevant expert meetings and forums. ECHOES was also cross-promoted through a page delivered by the EMIS-2017 survey, which was launched at the same. This page used the same screening questions as ECHOES to identify if EMIS responders were also CHWs and, if so, to then direct respondents to the ECHOES survey.

### Inclusion and Exclusion Criteria

The CHWs who satisfied the following criteria were eligible to participate in the survey if they (1) provided sexual health support for gay, bisexual, and other MSM in a community setting (not in a hospital or clinic) during the last 12 months; (2) provided support as a CHW in one of the 36 eligible countries (all 28 EU countries and neighbor countries: Bosnia Herzegovina, Iceland, Moldova, Norway, Russia, Serbia, Switzerland, and Ukraine); (3) were aged 18 years or older; and (4) consented to take part in the survey.

**Questionnaire Development**

The ECHOES survey was developed primarily by a Brighton-based study team of five academics: three psychologists specializing in MSM issues, behavioral medicine, survey design, and sexual health/HIV (NS, JH, CL); a social geographer with expertise in sexual and gender identities (NMG); and a former CHW/researcher (AP) in collaboration with colleagues from the wider ESTICOM project (particularly Objective Two partners OP, MK, MD, NL, CF, JC).

Before the survey was designed, a Europe-wide scoping exercise was conducted by the ESTICOM partners to review the extant literature regarding the knowledge, attitudes, and practices of CHWs concerning the sexual health of gay, bisexual, and other MSM [22]. Another more informal review was conducted by the ECHOES development team to develop a working definition of CHWs for European contexts and to explore the existence of any CHW surveys in Europe or elsewhere. An additional aim of this extra review was to consult with project partners to share any available national or regional questionnaires targeting CHW in any language. No national or regional questionnaires targeting CHW were submitted to the ECHOES development team. The outcomes of both scoping reviews were broadly consistent in showing a lack of both peer-reviewed and grey literature on CHWs involved in providing sexual health support aimed at gay, bisexual, and other MSM in Europe.

**Figure 1:**
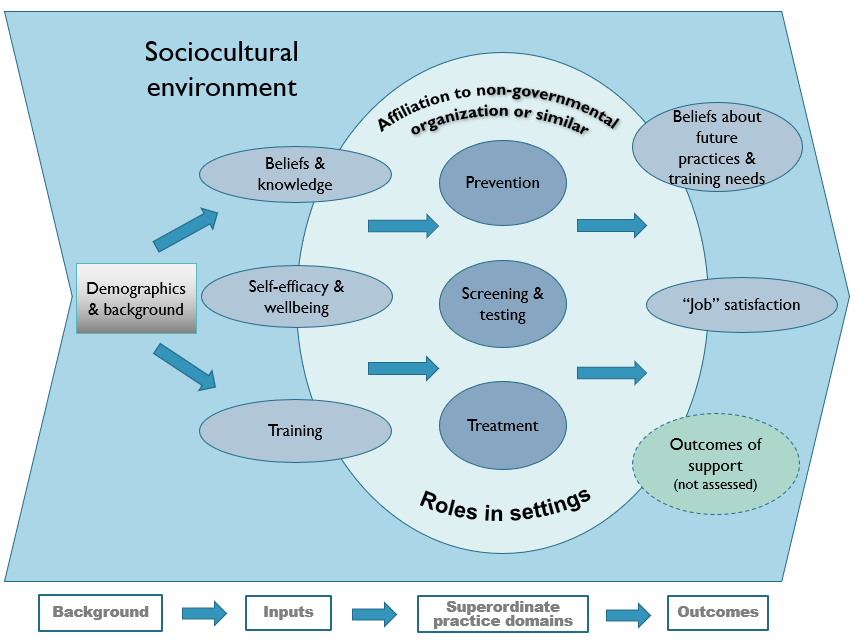
Diagram of ECHOES conceptual model.

In parallel to the scoping activities, an initial conceptual model of the survey was devised drawing on a consensus-building exercise with project partners to collate their views as experts on a number of issues, including screening (who to include and exclude), the relative importance of different proposed areas of interest for the CHW survey (demographics, CHW activities and roles, settings, motivations, attitudes, knowledge, barriers, CHW development and support, training needs, and open text to propose any additional area), and estimates of the extent of data to be collected. Figure 1 shows the final conceptual model underlying the ECHOES survey, including all major components captured by the questionnaire. The conceptual underpinnings of the survey are informed broadly by ideas coming from the theory of planned behavior [23,24] and other conceptual frameworks, such as the health belief model [25], which suggest that action is strongly influenced by beliefs about benefits (and costs) of activities and barriers and facilitators.

Based on the conceptual model, the questionnaire was structured around three superordinate practice domains of prevention, screening and testing, and treatment that form the core of the questionnaire (center of the model). These practice domains were shaped by (1) affiliation to organizations (NGO or similar) and (2) roles adopted in settings (eg, peer supporter, clinician working as a CHW within the community). Demographics, background variables, cognitions (beliefs and knowledge on HIV and other STIs prevention, screening and treatment), person variables (self-efficacy and well-being), and prior training and continuing professional development are inputs that shape practices. Beliefs regarding future practices (eg, providing community-based voluntary counseling and testing) and training needs, job satisfaction, and outcomes in MSM (not measured) reflect on and are a reflection of the activities carried out by CHWs (“practices”). For the purposes of this paper, they are considered outcomes.

The ECHOES conceptual model will most likely aid analysis but should not be seen as capturing causal relationships. Based on our global or systems perspective and the evidence available, links exist between many elements and influences are frequently bidirectional and probably recursive. The conceptual model will inform the statistical analysis, but given the provisional and conceptual nature of this model, it will neither determine nor limit the analysis to links proposed by the model.

### Piloting

Following the development of the conceptual model, the first full draft of the survey was developed on paper and online via Demographix in early 2017. A pretesting phase was initiated to make an initial assessment of this draft survey. Subsequent iterative rounds of small-scale online prepiloting were undertaken in February and March 2017, both informally and internally at the University of Brighton, as well as externally with CHWs known to the research team. Approximately 25 individuals participated in this pretesting phase, the purpose of which was to test out discrete sections of the questionnaire as they became available, checking for acceptability, completeness, comprehension, phrasing, and ease of use. As part of this process, respondents were asked to attempt to answer the draft sections followed by feedback to add, adapt, or delete questions as necessary to make them relevant to the target sample.

Following the completion of the series of online pretests, a broader consultation exercise was conducted using ESTICOM’s wider networks. The draft ECHOES survey was sent out for its first consultation via MailChimp to 412 unique email addresses of ESTICOM subscribers from March to April 2017. Twenty-eight detailed responses were received from 18 countries representing 25 organizations, including European agencies, national government departments, and specialist NGOs (eg, in sexual health, HIV, and LGBTI issues), community-based voluntary counseling and testing, public health agencies, and other organizations. The consultation provided a very clear steer on modifying the ECHOES survey to develop it further for online piloting and finalization. In responding to the outcomes of the consultation, every nomination for amendment (eg, cut, add, or change), comment, and criticism was considered by the ECHOES development team. Respondents identified typos and routing errors that were subsequently rectified. Discussion by the research team led to the deselection, modification, and the addition of numerous questions.

Following the pretesting phase, a small number (n=7) of cognitive debrief interviews were conducted by one of the authors (NMG) with participants experienced in CHW work and volunteering or appropriate fields of sexual health; recruitment was opportunistic, but heterogeneity was maximized. The aim of these interviews was to gather a rich evidence base to assess and improve the clarity, intelligibility, accessibility, and acceptability of the online survey. Data generated from the interviews was used to revise further the online survey before the wider online piloting.

Following the cognitive debriefing interviews, final adjustments were made to the survey and transferred into Demographix for the launch of a second pilot survey. The aims of the second pilot survey were to test the ECHOES survey in its most complete form and to provide sufficient data for validity checking of particular questions. Recruitment for the pilot test aimed for a sample size of 50 with a spread across European regions; however, the pilot was available in English only. The limited sample size was fixed to prevent potential exhaustion of the CHW population. The second pilot survey was opened for responses during three weeks in June 2017. An invitation to complete the pilot survey was emailed using MailChimp, and consortium partners were also asked to circulate the invitation through their own relevant networks. Reminder emails were sent on June 15 and 19, 2017. Fifty-four responses were received. Preliminary analysis of these pilot data demonstrated that the survey appeared to work well technically and could generate data that could answer the research objectives.

### Final ECHOES Questionnaire Design and Content

**Figure 2:**
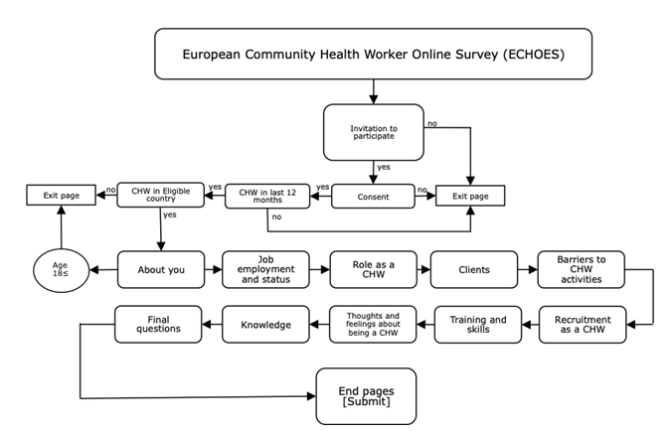
Flow diagram of ECHOES questionnaire structure.

With reference to Figure 1, the ECHOES survey comprised three superordinate domains, with 175 questions (heavily routed) divided into 10 subsections (see Figure 2); up to 250 data points were collected for each respondent. Approximately 10% of all questions were drawn from three validated scales documenting well-being, self-efficacy, and job satisfaction of CHWs [26-28]. The remaining 90% of questions were developed or adapted by the authors. The final survey was more than 27 pages, 13 of which contained core questions addressed to all respondents. Ten pages were conditional on the answers to preceding questions, and the remaining four were exit pages that showed when a participant was not eligible to complete the survey. Brief descriptions of each subsection of the questionnaire are provided subsequently; examples of questions for each section are provided in Textbox 1.

**Examples of ECHOES questions per section.**

**About you**

- Which of the following best describes how you think of yourself [gender identity response set]; ...is this what you were assigned at birth? [trans experience; y/n]
- Which of the following best describes how you think about yourself? (sexual orientation response set); Thinking about all the people who know you (including family, friends, and work or study colleagues), what proportion know this? [outness response set]

**Job employment and status**

- We know that many people do not use the term “community health worker.” How would you describe your job title? [free-text]
- When working as a CHW, which of the following best describes the type of organization you work for/with? [organization response set]

**Role as a CHW**

- Tick all that apply:
  - For the purposes of prevention, I am involved in providing information about... [information response set (eg, safer sex, testing, vaccinations, chemsex)]
  - I am involved in providing these intervention activities......[intervention response set (eg, supporting use of PreP/PEP, sexual health provision, mental health support)]
  - Where do you deliver prevention activities around HIV/AIDS, viral hepatitis and STIs to gay, bisexual and other MSM? [settings response set (eg, gay venues)]

**Clients**

- Which three of these populations of people do you most often work with in your CHW activities? [population response set]
- Thinking only about your work with gay, bisexual and other MSM regarding delivering sexual health support on HIV, viral hepatitis and other STIs, what age group do you most often work with? [age response set]

**Barriers to CHW activities**

- Think about all the activities you do in your role as a CHW. Please tick the main issues for you as an individual which hinder your activities [individual barriers response set]
- Please tick the main issues from your organization which hinder your activities [organizational barriers response set]

**Recruitment as a CHW**

- Why did you start to work/volunteer as a CHW? [motivation response set]
- How did you first become a CHW?

**Training and skills**

- Thinking about your current role as a CHW, have you received training in this role? If yes—What kind of training have you received? [training type response set]
- In order to be as effective as possible in your current role, which areas would you most benefit from additional training in? [training areas response set]

**Thoughts and feelings about being a CHW**

- Please think about your day to day life, including your role as a CHW. How true are the following statements? [true/not true response set]:
  - It is easy for me to stick to my aims and accomplish my goals.
  - I am confident that I could deal efficiently with unexpected events.
  - Thanks to my resourcefulness, I know how to handle unforeseen situations.
- Taking everything into consideration, how do you feel about your activities as a CHW as a whole? [satisfaction response set]

**Knowledge**

- Regarding HIV/AIDS and hepatitis B and C, how confident are you in your knowledge of... prevention; screening and/or testing; treatment and/or support? [HIV/AIDS/ Hepatitis B and C, confidence response set]

**Final questions**

- Have you ever been diagnosed with HIV?
- How good is your health in general? [general health status response set]
- Please indicate which is closest to how you’ve been feeling over the last two weeks: [time response set]:
  - I have felt cheerful and in good spirits
  - I have felt calm and relaxed
  - I have felt active and vigorous
  - I woke up feeling fresh and rested
  - My daily life has been filled with things that interest me

#### About You

The ECHOES survey is part of the larger EU ESTICOM project and was intended to sit alongside the EMIS 2017 survey; therefore, the demographic indicators were harmonized between the two surveys as much as possible. This section contained a total of 11 questions covering age, gender identity (inclusive of trans and gender-nonconforming identities), sexual identity (orientation), “outness,” membership within an ethnic or racial minority, location of CHW activities, years in full-time education since the age of 16 years, perception of household income, and languages spoken (native and other). Linking with the final survey section (see Final Questions section), some of these items also assessed peer status; namely, whether the CHWs share characteristics with the populations they serve.

#### Job Employment and Status

This section asked about the CHW job role (paid or unpaid) in providing sexual health support to gay, bisexual, and other MSM. If not currently employed as a CHW, respondents were asked to answer about their most recent CHW role in the last 12 months. Given that “CHW” is an unfamiliar term in Europe, the first question asked participants to describe their job role (open question). Additional questions included employment status and job security (if part time, additional questions on status when not working as a CHW), and affiliated organization (if any) including its main purpose, size, and funding sources.

#### Role as a Community Health Worker

One of the key aims of the ECHOES survey was to find out what CHWs actually do. Therefore, this section asked respondents about their personal involvement in CHW activities over the last 12 months relating to sexual health support to gay, bisexual, and other MSM around HIV/AIDS, viral hepatitis, or other STIs. The cognitive debriefing interviews highlighted that CHWs work in a wide variety of organizations, beyond organizations specializing in gay, bisexual, and MSM sexual health and/or HIV/AIDS. Given the complexity of these CHW roles (practices) within diverse contexts, the wording of questions and data items throughout this section (and the wider survey) were designed to capture responses from those who have a CHW role as part of their wider job, those who volunteer unpaid, those who do not currently have a CHW role but did within the past 12 months, and those whose CHW role involved gay, bisexual, and MSM as well as those who did not fall into this grouping (eg, heterosexual men, women). This section comprised three large subsections, including prevention, screening and/or testing, and treatment and/or support, with each subsection containing multiple items. Each subsection covered specific CHW activities and their frequency as well as the settings in which they occur.

#### Clients

This included the people CHWs work with and their relationships with them. This section asked which populations the CHW worked with most often, including their approximate age band (<25 years, >25 years, even mix), how many clients they see in a seven-day period, and their perceptions of client trust in their support and associate organization (if relevant).

#### Barriers to Community Health Worker Activities

This section consisted of six questions about the issues that shaped or hindered their role and activities as a CHW at different levels (individual, organizational, societal/cultural), including how things might be improved.

#### Recruitment as a Community Health Worker

These eight items refered to how CHWs were recruited to their post when they first started as a CHW or first became involved with activities supporting gay, bisexual, and other MSM, including whether training, qualifications, and/or experience were required.

#### Training and Skills

A key part of the ECHOES survey was to identify training needs to inform the third objective of the ESTICOM project (development of a specific training program for CHWs). In this section, 11 questions explored training received, intensity (amount), on-going or not, and topic areas covered, including who identified and paid for the training, whether training was allowed in work time, and requirements (and priority) for future training.

#### Thoughts and Feelings About Being a Community Health Worker

This section included two validated scales, including (1) an adapted and shortened general self-efficacy scale (6 items) by Romppel et al [26] based on Schwarzer et al [27], and (2) a 10-item shortened version scale similar to Goetz et al [28] to assess job satisfaction including a global job satisfaction rating.

#### Knowledge

For practical purposes and because the ECHOES survey is designed to inform training needs, knowledge of HIV/AIDS, viral hepatitis, and other STIs as a CHW was assessed in terms of confidence judgments regarding core knowledge domains. CHWs were asked to rate how confident they were in their knowledge of HIV/AIDS, viral hepatitis, and STIs on a scale from 1 (not confident at all) to 5 (very confident) in three different areas drawing on self-efficacy theory [29]: (1) prevention, (2) screening and/or testing, and (3) treatment and/or support (for a practical example see [30]).

#### Final Questions

The final 12 questions of the survey were designed to understand how CHWs may be connected to the communities they serve. Five of these comprised the WHO-5 Brief Well-Being Index [31] to assess general well-being and/or good emotional and positive aspects of mental health. A single question assessed overall health, and the remaining questions assessed aspects relating to whether CHWs share some characteristics with the populations they serve (eg, living with HIV, drug use).

### Translation and Sociolinguistic Equivalence

To facilitate translation, the Demographix platform provided a custom interface for the translation of the signed-off English language version of the ECHOES questionnaire to all required languages. The interface allowed translators to enter the survey via a unique and personalized URL and to see a locked version of the original English version on the left of their screen while translating the survey directly over the top of a second version of the English original, on the right of their screen. Using this service ensured that all questions maintained the same routing and piping instructions in all languages, and all versions were structurally identical. Demographix also provided existing pretranslated survey completion instructions (eg, next, previous, submit) in all the required languages for ECHOES.

Multilingual proofreaders were asked to use a similar system to compare and contrast survey translations. Demographix also allowed simultaneous access to all ECHOES partners who needed to review a specific version of the survey before being published and launched. Translations were outsourced to translators suggested by the project’s collaborating partners, thereby minimizing costs. Translations involved native-speaking stakeholders from the field (ie, experts in HIV prevention or LGBT health) as translators for each language. Two multilanguage proofreaders were involved when possible to compare the translations with the English original but also with one another. The proofreaders ensured a harmonized, multilanguage questionnaire, while deliberately maintaining certain differences identified as culturally appropriate, such as explicitness of language or the question of formal or informal address.

In ECHOES, the standardized scales used came with existing translations. The generalized self-efficacy scale and the WHO-5 Brief Well-Being Index were available in all languages required for the survey. The job satisfaction scale was available in English and German. Translators were asked to use the already translated versions when possible, and if translations did not exist, to provide their own translation.

The final questionnaire was available in the following 14 EU languages: Bulgarian, Croatian/Serbian, Czech, Dutch, English, Finnish, French, German, Greek, Italian, Polish, Portuguese, Romanian, and Spanish. ECHOES was also translated into Russian, because it is a major ethnic minority language, and into Ukrainian. After consultation with Scandinavian (Norway, Sweden, Denmark) and Baltic (Estonia, Latvia, Lithuania) country representatives, it was decided not to translate the ECHOES questionnaire into these languages because the few expected CHW in these countries were assumed to be able to understand and complete the English- or Russian-language questionnaires. Therefore, ECHOES was available in 16 languages in total.

### Data Analysis and Management

In general, data analysis will be exploratory, although we will be exploring some issues in line with existing research findings. This includes a gradient across Europe (west to east) of intensifying stigma and discrimination. Scale scores will be created for the standardized instruments following published procedures. To ensure internal consistency of scales for the sample in this survey, internal consistency reliability will be checked with Cronbach alpha. Descriptive findings will be reported as means and standard deviations for continuous variables, and as numbers and percentages for categorical variables. Descriptive analyses will be run in SPSS using the overall ECHOES dataset, including all language versions of the ECHOES questionnaire. Bivariate analysis, including chi-square tests (or Fisher exact test when appropriate) and Mann-Whitney *U* tests, will be used to determine significant differences between groups, for categorical variables including demographics. Kruskal-Wallis tests will be used for continuous variables.

Only the ECHOES development team at the University of Brighton and the data analysis team at the Centre d’Estudis Epidemiològics sobre les Infeccions de Transmissió Sexual i Sida de Catalunya (CEEISCAT) in Badalona, Spain, will have access to the data during the study. After the study is completed, the University of Brighton and CEEISCAT will make the relevant data available to consortium partners for analysis as appropriate.

### Ethics

Ethical approval for the initial questionnaire design and development activities (eg, cognitive debrief interviews, pretesting, piloting) was obtained from the University of Brighton’s School of Health Sciences, School Research Ethics and Governance Panel. Additional approval to host the survey online and recruit participants was received from the Hospital Universitari Germans Trias i Pujol in Badalona, Catalonia (Spain) (PI-16-143), as the hosting institution of CEEISCAT.

#### Informed Opt-In Consent

Respondents who accepted the invitation to take part in the ECHOES study and used the link provided to access the survey Web page were taken to the survey introductory page. Participants were then provided with information about the project, confidentiality of the survey findings, and an outline of what participants were required to do and how long it would take to complete the questions. A statement was provided regarding data protection, including confidentiality and anonymity as well as a brief statement about the ESTICOM project consortium. Potential participants were asked to click on a box to confirm that they had read and understood the participant information before proceeding, a box to confirm that they understood their participation would be voluntary and that they would be able to withdraw at any time, and a box explicitly requesting them to opt-in, thus confirming their agreement to take part in the survey.

#### Confidentiality

No personal data (such as names, addresses, date of birth) were collected from participants. The survey was completely anonymous; no IP addresses were stored or downloaded and no information regarding the origin of the “‘click” was collected. No cookies were installed on the potential participant’s computer or device.

### Planned Dissemination

The results of the ECHOES survey will be published in consortium reports submitted to the Chafea, in peer-reviewed scientific journals, and via conference presentations. Results of the study will also be disseminated through the ESTICOM network via MailChimp and supported by AIDS Action Europe, as well as on the ESTICOM project website.

## Results

The ECHOES survey (part of the ESTICOM project) is funded by Chafea of the European Commission. Survey enrollment closed on January 31, 2018. A total of 1181 participants responded to the survey. Responses were screened for key inclusion criteria. Those who did not deliver services to MSM in a community setting (n=107), work or were active in the countries included in the study (n=24), or meet the minimum age of 18 years (n=15) were excluded, resulting in a final sample available for analysis of 1035 CHWs. The findings of the ECHOES survey and the wider ESTICOM project, are now available from the ESTICOM website and/or by contacting the first author.

## Discussion

To our knowledge, this study is the first internet-based self-completion questionnaire survey exploring the knowledge, attitudes, and practices of CHWs providing sexual health support to gay men, bisexual men, and other MSM in European settings. It is expected that the results will transform our understanding of who CHWs in Europe are; what they do; where, how, and why they do what they do; as well as identify the individual, organizational, and structural barriers and challenges to CHWs’ activities. By gaining a deeper understanding of CHWs’ knowledge, attitudes, and practices regarding their clients, and given that ECHOES is part of the much larger ESTICOM project that includes the EMIS-2017 survey, findings are also expected to generate insights for the development of the first European common training program for CHWs (aim 3 of ESTICOM). We expect this impact to be considerable, with findings highlighting important areas to strengthen and build the capacity of CHWs in all the 36 ECHOES-eligible countries (all 28 EU countries and neighbor countries, including Bosnia Herzegovina, Iceland, Moldova, Norway, Russia, Serbia, Switzerland, and Ukraine).

The questionnaire will also garner information about profile characteristics of CHWs, which may be important in supporting CHWs, allowing them to develop their professional profile and informing of psychosocial training needs. This will be supported by information on both general and emotional health, job satisfaction, and acceptance of gay, bisexual, and MSM people.

The pan-European nature of this study will provide a comprehensive dataset across participating countries that will enable analysis of variability observed in CHWs’ knowledge, attitudes, and practices. As the output of a European Commission tender, it is anticipated that this knowledge of the variability among CHWs along with insights for the development of common training will be important in the development of future policy initiatives around promoting health, reducing new infections, and ultimately working toward global Sustainable Development Goals (goals 3, 10, and 11) and achieving the UNAIDS 90/90/90 targets.

## Abbreviations

Chafea: Consumers, Health, Agriculture and Food Executive Agency
CHW: community health worker
ECHOES: European Community Health Worker Online Survey
EEA: European Economic Area
EU: European Union
MSM: men who have sex with men
NGO: nongovernmental organizations
STI: sexually transmitted infections
WHO: World Health Organization

## References

[1] Elford J, Hart G, Sherr L, Williamson L, Bolding G (2002). Peer led HIV prevention among homosexual men in Britain. Sex Transm Infect.

[2] Kelly JA (2004). Popular opinion leaders and HIV prevention peer education: resolving discrepant findings, and implications for the development of effective community programmes. AIDS Care.

[3] (2001). American Public Health Association.

[4] Lefeuvre D, Dieng M, Lamara F, Raguin G, Michon C (2014). Community health workers in HIV/AIDS care. Sante publique.

[5] Goodwin K, Tobler L (2008). Community Health Workers: Expanding the Scope of the Health Care Delivery System.

[6] Ingram M, Reinschmidt KM, Schachter KA, Davidson CL, Sabo SJ, De Zapien JG, Carvajal SC (2012). Establishing a professional profile of community health workers: results from a national study of roles, activities and training. J Community Health.

[7] Mel Zuckerman, Enid Zuckerman National Community Health Worker Advocacy Survey. Preliminary Data Report for the United States and Territories.

[8] Mwai G, Mburu G, Torpey K, Frost P, Ford N, Seeley J (2013). Role and outcomes of community health workers in HIV care in sub-Saharan Africa: a systematic review. J Int AIDS Soc.

[9] Nyamathi A (2014). engaging community health workers in HIV/AIDS care: a case exemplar among rural Indian women living with AIDS. J HIV/AIDS Soc Serv.

[10] Schneider H, Hlophe H, van Rensburg Dingie (2008). Community health workers and the response to HIV/AIDS in South Africa: tensions and prospects. Health Policy Plan.

[11] Sabo S, Allen C, Sutkowi K, Wennerstrom A (2017). Community health workers in the United States: challenges in identifying, surveying, and supporting the workforce. Am J Public Health.

[12] National Institute for Health and Care Excellence (2016). Community Engagement: Improving Health and Wellbeing and Reducing Health Inequalities.

[13] Olaniran A, Smith H, Unkels R, Bar-Zeev S, van den Broek N (2017). Who is a community health worker? – a systematic review of definitions. Global Health Action.

[14] World Health Organization (2016). Global Strategy on Human Resources for Health: Workforce 2030.

[15] European Centre for Disease Prevention and Control/WHO Regional Office for Europe (2018). HIV/AIDS Surveillance in Europe 2018-2017 Data.

[16] Batchelder AW, Safren S, Mitchell AD, Ivardic I, O'Cleirigh C (2017). Mental health in 2020 for men who have sex with men in the United States. Sex Health.

[17] (2007). Outreach Work Among Marginalised Populations in Europe: Guidelines on Providing Integrated Outreach Services.

[18] South J, Meah A, Bagnall A, Kinsella K, Branney P, White J, Gamsu M (2010). People in Public Health–A Study of Approaches to Develop and Support People in Public Health Roles.

[19] South J, Jackson Kl, Warwick-Booth L (2011). The community health apprentices project–the outcomes of an intermediate labour market project in the community health sector. Community Work Fam.

[20] Lehman U, Sanders D (2007). Health Workers: What Do We Know About Them? The State of the Evidence on Programmes, Activities, Costs and Impact on Health Outcomes of Using Community Health Workers.

[21] Sherriff N, Huber J, McGlynn N, Llewellyn C (2017). A Final Proposal for a European Community Health Worker Survey (ECHOES).

[22] Folch C, Fernández-Dávila P, Palacio-Vieira J, Dutarte M, Maria-Corbelli G, Block K (2017). A Review of Community Health Worker (CHW) Knowledge, Attitudes and Practices Relating to the Sexual Health of MSM, Including Existing Training Materials and Manuals in Europe and Neighbouring Countries (D5.1).

[23] Ajzen I (1991). The theory of planned behavior. Organ Behav Hum Dec.

[24] Ajzen I, Fishbein M (1980). Understanding Attitudes and Predicting Social Behavior.

[25] Becker M, Maiman LA (1975). Sociobehavioral determinants of compliance with health and medical care recommendations. Med Care.

[26] Romppel M, Herrmann-Lingen C, Wachter R, Edelmann F, Düngen HD, Pieske B, Grande G (2013). A short form of the General Self-Efficacy Scale (GSE-6): development, psychometric properties and validity in an intercultural non-clinical sample and a sample of patients at risk for heart failure. Psychosoc Med.

[27] Schwarzer R, Jerusalem M, Weinman J, Wright S, Johnston M (1995). Generalized Self-Efficacy scale. Measures in Health Psychology: A User's Portfolio. Causal and Control Beliefs.

[28] Goetz K, Campbell S, Steinhaeuser J, Broge B, Willms S, Szecsenyi J (2011). Evaluation of job satisfaction of practice staff and general practitioners: an exploratory study. BMC Fam Pract.

[29] Bandura A (1977). Self-efficacy: toward a unifying theory of behavioral change. Psychol Rev.

[30] Hecimovich MD, Styles I, Volet SE (2014). Development and psychometric evaluation of scales to measure professional confidence in manual medicine: a Rasch measurement approach. BMC Res Notes.

[31] Bech P (1998). Quality of Life in the Psychiatric Patient.

